# A Hierarchical Feature and Sample Selection Framework and Its Application for Alzheimer’s Disease Diagnosis

**DOI:** 10.1038/srep45269

**Published:** 2017-03-30

**Authors:** Le An, Ehsan Adeli, Mingxia Liu, Jun Zhang, Seong-Whan Lee, Dinggang Shen

**Affiliations:** 1 Department of Radiology and Biomedical Research Imaging Center (BRIC), University of North Carolina at Chapel Hill, NC 27599, USA; 2 Department of Brain and Cognitive Engineering, Korea University, Seoul 02841, Republic of Korea

## Abstract

Classification is one of the most important tasks in machine learning. Due to feature redundancy or outliers in samples, using all available data for training a classifier may be suboptimal. For example, the Alzheimer’s disease (AD) is correlated with certain brain regions or single nucleotide polymorphisms (SNPs), and identification of relevant features is critical for computer-aided diagnosis. Many existing methods first select features from structural magnetic resonance imaging (MRI) or SNPs and then use those features to build the classifier. However, with the presence of many redundant features, the most discriminative features are difficult to be identified in a single step. Thus, we formulate a hierarchical feature and sample selection framework to gradually select informative features and discard ambiguous samples in multiple steps for improved classifier learning. To positively guide the data manifold preservation process, we utilize both labeled and unlabeled data during training, making our method semi-supervised. For validation, we conduct experiments on AD diagnosis by selecting mutually informative features from both MRI and SNP, and using the most discriminative samples for training. The superior classification results demonstrate the effectiveness of our approach, as compared with the rivals.

Computer-aided diagnosis often involves decision making using computer algorithms^1^. For example, disease can be identified by machine learning tools, such as classification models^2^. Design of automated classification algorithms is highly imperative, in order to provide physicians with a second opinion for more accurate diagnosis. The quality of computer-aided diagnosis relies on the trained classifiers. To learn such classifiers, annotated samples, each of which contains a number of features, are utilized in the training process. Ideally, only informative features and discriminative samples shall be used for effective learning.

For a concrete example, as one of the most common neurodegenerative diseases found in elderly, Alzheimer’s disease (AD) accounts for up to 70% of dementia cases^3^. As AD is a progressive disease which affects memory and other important mental functions, its symptoms gradually deteriorate over time. With increased human life expectancy, growing numbers of elderly are likely to suffer from dementia. It is estimated that by 2050, one new case of AD will occur every 33 seconds, and the total population affected is expected to reach 13.8 million^4^. Unfortunately, thus far, there is no effective cure for AD^5^. The early stage of AD is commonly referred to as mild cognitive impairment (MCI). During disease progression, a healthy normal control (NC) may first develop MCI, and then worsening symptoms result in AD. A previous study indicated that MCI patients progressed to AD at a yearly rate of 10% to 15%^6^. Since there is no clear rule to discern AD, NC, and MCI, accurate AD and early stage MCI diagnoses are very challenging obstacles. Nevertheless, once AD or MCI is diagnosed, early treatment including medications and management strategies could help improve symptoms^7^,^8^. Therefore, timely and accurate diagnoses of AD and MCI are highly desirable.

Among various diagnosis tools, brain imaging modalities, such as structural magnetic resonance imaging (MRI), have been extensively utilized due to their accurate measurements of brain structures, especially in the hippocampus and other AD related regions^9^,^10^,^11^,^12^,^13^,^14^,^15^,^16^. Based on differences in brain shape and neuroanatomical configuration, brain imaging techniques help identify abnormal brain structures in those with AD or MCI. When multiple atlases or templates are available, the classification performance can be further improved^17^,^18^. Besides structural MRI, other imaging modalities such as functional MRI can also be used in AD/MCI diagnosis^19^,^20^,^21^,^22^,^23^, as they provide additional functional information about hypometabolism and specific protein quantification, which can be beneficial in disease diagnosis.

Besides imaging data that provide tissue level information to help AD diagnosis, genetic variants, which are related to AD, have also been shown to be valuable for AD diagnosis^24^,^25^. Genome-wide association studies (GWAS) were conducted to discover the association between the single nucleotide polymorphism (SNP) and the imaging data^26^. The SNP reveals molecular level information, which is complementary to the tissue level information in the imaging data. In ref. 27, the associations between SNPs and MRI-derived measures with the presence of AD were explored and the informative SNPs were identified to guide the disease interpretation. To date, most previous works have focused on analyzing the correlation between imaging and genetic data^28^, yet using both types of data for AD/MCI diagnosis has received very limited attention^29^.

Computer-aided diagnoses, including those for AD/MCI, often encounter a challenge that the data dimensionality is usually much higher than the number of available samples for model training^30^. This imbalance between feature number and sample size may affect the learning of a classification model for disease prediction, or a regression model for clinical score prediction. Furthermore, feature redundancy exists in both imaging and genetic data in terms of specific diseases. For example, in MRI-based diagnosis, features are usually generated by segmenting a brain into different regions-of-interest (ROIs)^29^. As some of the ROIs may be irrelevant to AD/MCI, feature selection can be conducted to identify the most relevant brain regions in order to learn the classification model more effectively. Similarly, only a small number of SNPs from a large pool are associated with AD/MCI^29^. Therefore, it is preferable to use only the most discriminative features from both MRI and SNPs for classification model training.

For AD diagnosis, various feature selection schemes, either unsupervised or supervised, have been proposed. Typical feature selection methods include *t*-test^31^, Fisher score^32^, Laplacian score^33^, and Pearson correlation^34^. Recently, sparse feature learning, *e.g*., the LASSO-based sparse feature learning^35^, has become a popular choice for feature selection. Besides using the 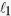-norm based sparsity constraint for feature selection, the grouping or relational information embedded in data has also been introduced for improving feature selection procedures^17^,^36^. It is also important to mention that the unsupervised methods often consider certain data distributions or manifold structures, while the association between features and the corresponding class labels are overlooked. On the other hand, the supervised feature selection methods can be more effective by utilizing the label information in the learning process. In practice, unlabeled data may also be available but unusable by the supervised methods. In addition, while most of the previous works focused on feature selection, they did not consider discarding poor samples. Those unwanted samples may have been contaminated by noise, or may be outliers. Including poor samples can affect the model learning, thus degrading the diagnosis accuracy^37^.

In this paper, we propose a joint feature and sample selection framework which takes advantage of all labeled data along with unlabeled ones, in order to find the most informative features for classifier training. Specifically, a semi-supervised hierarchical feature and sample selection (ss-HMFSS) framework is introduced, which simultaneously selects discriminative features and samples from multimodal data. Besides a sparse constraint, we also impose a manifold term, which regularize on both labeled and unlabeled data. This regularization term preserves the neighborhood structures during the mapping from the original feature space to the label space. In our semi-supervised setting, we are able to exploit useful information from both labeled and unlabeled data, wherein the latter of which may be abundant in clinical practice.

Since the redundant features and poor samples may not be scarce, instead of achieving feature and sample selection in one single step, we perform feature and sample selection in a hierarchical manner, *i.e*., in multiple steps. Moreover, the feature coefficients learned in one hierarchy are used not only to discard unimportant features but also to weight the remaining features. The updated features and pruned sample set from each current hierarchy are supplied to the next round to further identify a smaller subset with even more discriminative features and samples. In this way, we gradually refine the feature and sample sets step-by-step, undermining the effect of non-discriminative data.

To validate our methodology, we conduct experiment on AD diagnosis. Structural MRI and SNPs are jointly used to improve the diagnosis accuracy, as data from both modalities are mutually informative measures in understanding disease prognosis^26^. The final selected features and samples by our method are used to train classifiers (in our case we use a Support Vector Machine (SVM)). The experimental data include 737 subjects from the Alzheimer’s Disease Neuroimaging Initiative (ADNI) cohort. In different classification tasks, *i.e*., AD versus NC, MCI versus NC, and progressive MCI (pMCI) versus stable MCI (sMCI), our method demonstrates superior results, as compared with other competing methods.

## Results

### Experimental Settings

We consider three binary classification tasks in the experiments, namely AD vs. NC, MCI vs. NC, and pMCI vs. sMCI. We adopt a cross-validation strategy (10-fold) in order to examine the classification performance. In detail, the data are randomly divided into ten roughly equal portions, and in each fold, the subjects in one fold are used as testing data, while the rest subjects are used as training data. Such process is executed ten times to alleviate bias in random partitioning. For the unlabeled data in our method, we choose the irrelevant subjects with respect to the current classification task, *e.g*., when we classify AD and NC, the data from MCI subjects are used as unlabeled data. The dimensionality of the SNP features is reduced to that of the MRI features before our joint feature and sample learning.

The parameters in feature and sample selection for each classification task are selected by grid search on the training data. The parameters *λ*_1_ and *λ*_2_ in Eq. 7 for regularization purpose are searched from the range {2^10^, 2^−9^, …, 2^0^}. After each hierarchy, 5% samples are discarded, and the features whose coefficients in **w** are smaller than 10^−3^ are removed. The neighborhood size *K* in Eqs (5) and (6) is set to 20, as we empirically find that this is a reasonable choice to allow sufficient neighbors to assign a reliable soft label to unlabeled samples. To train the classifier, we use the implementation of LibSVM^38^ for linear SVM model training with the default parameter *C* = 1, since we observe that the results are not sensitive to the changes in this parameter. To validate the statistical significance of our method, we perform paired-sample *t*-test to compare our method with the other benchmark methods.

### Effects of Hierarchical Structure

**Figure 1:**
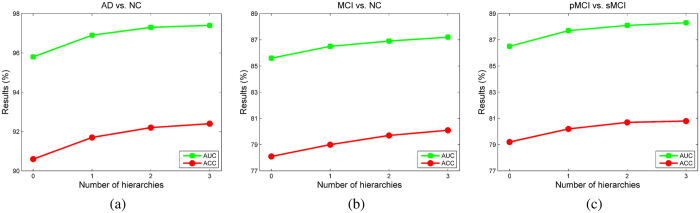
Effects of using different numbers of hierarchies. (**a**) AD vs. NC. (**b**) MCI vs. NC. (**c**) pMCI vs. sMCI.

To examine the effectiveness of the proposed hierarchical framework, Fig. 1 compares the classification accuracy (ACC) and area under the receiver operating characteristic (ROC) curve (AUC) under different settings of number of hierarchies. It is observed that the use of more hierarchies benefits the classification performance in all tasks, although the improvement becomes marginal after three hierarchies. Especially for the task of pMCI vs. sMCI classification, where the training data are not abundant, keeping discarding samples and features in a sequence of hierarchies may result in insufficient classification model learning. Therefore, we set the number of hierarchies to three in the following experiments. After this iterative process, on average, about 40% of the features are selected for training the classification models. It is also worth mentioning that compared to AD vs. NC classification, MCI vs. NC and pMCI vs. sMCI classifications are also critical in early diagnosis and possible therapeutic interventions, and these tasks can be more difficult, as demonstrated by lower values in ACC and AUC.

### Effects of Multimodal Features

**Figure 2:**
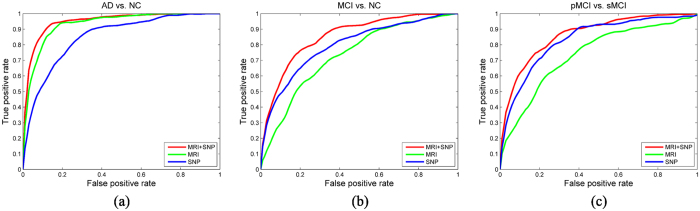
ROC curves of using different feature modality. (**a**) AD vs. NC. (**b**) MCI vs. NC. (**c**) pMCI vs. sMCI.

In our method, both MRI and SNP features are used. To study the contribution of individual feature modality, the ROC curves of the classification results using single feature modality are compared with those using both modalities in Fig. 2, and the values of ACC and AUC are listed in Table 1. As observed, using both modalities, *i.e*., MRI and SNP, better classification performances are achieved as compared with the use of a single feature modality in different classification tasks. This suggests that our method can effectively utilize the information from both modalities, and therefore produces better overall performance. For AD vs. NC classification, MRI features are more discriminative than the SNPs, while the opposite is observed in MCI vs. NC and pMCI vs. sMCI classifications. This suggests that the SNP features are more helpful in discerning the subtle differences in the possible presence of MCI.

### Effects of Feature and Sample Selection

In our method, we select both discriminative features and samples to help build better classification models. To verify the individual contribution of feature and sample selection, we compare the ACC and AUC values of the proposed method with both feature and sample selection, to using only sample or feature selection. The outcomes are shown in Table 2. We can observe that when using sample selection or feature selection only, the classification performance is inferior to the proposed method with both sample and feature selection. The contribution of feature selection is more significant than that of sample selection. This suggests that removing feature redundancy is more imperative. It is worthwhile to mention that while discarding samples is helpful, excessively doing so may be less effective or even counterproductive, due to the small sample size for training as a result.

### Comparison with Other Methods

For a more comprehensive comparison, the proposed method (ss-HMFSS) is compared with some popular and advanced methods for AD related diagnosis. Specifically, the methods being compared are the following:

- No feature selection (no FS), using MRI features only.
- No feature selection, using SNP features only.
- No feature selection, using both MRI and SNP features.
- Laplacian score^33^.
- Pearson correlation^34^.
- *t*-test^31^.
- Fisher score^32^.
- LASSO-based sparse feature learning^35^.
- Feature selection by relationship induced multi-template learning (RIML)^17^.
- Proposed method without unlabeled data (HMFSS).
- Proposed method with unlabeled data (ss-HMFSS).

For all methods, linear SVM is used as the classifier. To more thoroughly compare performances, besides ACC and AUC, we also report sensitivity (SEN) and specificity (SPE). The sensitivity is defined by SEN = TP/(TP + FN) and the specificity is defined by SPE = TN/(TN + FP), in which TP denotes true positive, FN denotes false negative, TN denotes true negative, and FP denotes false positive. SEN measures the classification accuracy for the positive samples, and SPE measures the classification accuracy for the negative samples.

The average classification results from the 10-fold cross-validation are reported in Table 3. Regarding the overall performance, AD vs. NC classification is relatively easier for different methods, as compared with MCI vs. NC and pMCI vs. sMCI classifications, as evidenced by higher performances in AD vs. NC classification. Regarding feature modality, MRI is more discriminative than SNP in distinguishing AD from NC, while for MCI vs. NC and pMCI vs. NC classifications, SNP is more useful.

Directly combining features from two different modalities may not necessarily improve classification performance. For example, in AD vs. NC, simply concatenating MRI and SNP features decreases the classification accuracy to 87.5%, as compared to an accuracy of 88.3% by using only MRI features. This is because SNP features are less discriminative for this classification task, and simply adding them affects the classification model learning. When features from both modalities are combined, a feature selection step is helpful, as indicated by the improved results using different feature selection methods. Compared with unsupervised feature selection method such as Laplacian score^33^, the supervised ones, *i.e*., Fisher score^32^ and LASSO^35^ perform better.

The RIML method^17^ is a recently proposed multimodal feature selection method, representing the state-of-the-art in feature selection for AD and MCI diagnosis. It considers the relationships among samples and different feature modalities when performing feature selection in a single step. On the contrary, we improve the effectiveness of feature selection by employing a hierarchical framework to keep only the most discriminative features and samples for training classification models. Even without unlabeled data, our method (*i.e*., HMFSS) outperforms RIML^17^, with the accuracy improvements being 1.4%, 2.0% and 1.3% respectively for the three classification tasks. When unlabeled data are incorporated to facilitate the learning process, our method (*i.e*., ss-HMFSS) obtains even further improved results in terms of all different measures.

### Analysis of Selected Features

#### Selected MRI Features

**Figure 3:**
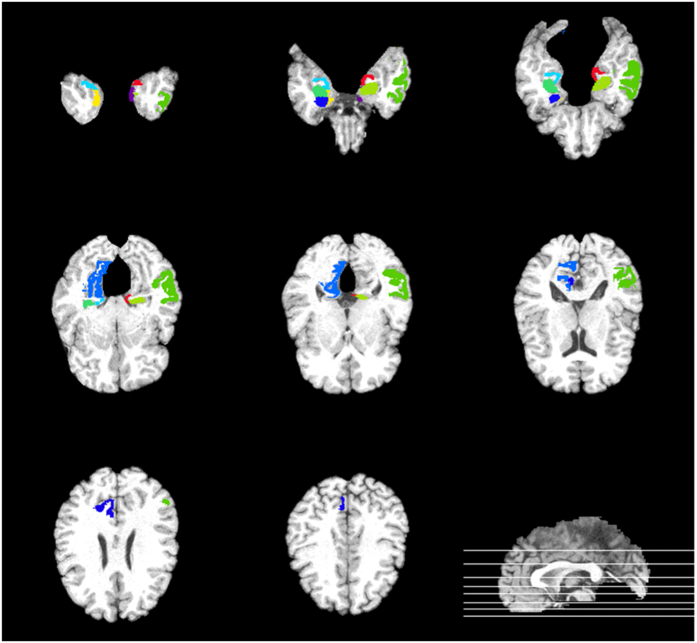
Top 10 most selected ROIs for AD diagnosis. These regions are (1) hippocampal formation left, (2) hippocampal formation right, (3) parahippocampal gyrus left, (4) parahippocampal gyrus right, (5) middle temporal gyrus left, (6) precuneus right, (7) entorhinal cortex left, (8) entorhinal cortex right, (9) medial occipitotemporal gyrus right, and (10) amygdala right.

In Fig. 3, we show the top 10 most discriminative ROIs for AD-related diagnosis in our method. Namely, those ROIs are (1) *hippocampal formation left*, (2) *hippocampal formation right*, (3) *parahippocampal gyrus left*, (4) *parahippocampal gyrus right*, (5) *middle temporal gyrus left*, (6) *precuneus right*, (7) *entorhinal cortex left*, (8) *entorhinal cortex right*, (9) *medial occipitotemporal gyrus right*, and (10) *amygdala right*. The features extracted from those ROIs are selected in the hierarchical process as most informative ones. Note that in previous studies, regions including hippocampal formation, parahippocampal gyrus, middle temporal gyrus, and precuneus, have been shown to be related to AD^39^,^40^,^41^. The selections of ROIs by our method are congruent with those from previous works.

#### Selected SNP Features

The most frequently selected SNP features and their gene origins are listed in Table 4. These genes have also been reported to be related to AD in previous works^29^,^42^,^43^,^44^. For example, the CTNNA3 gene, which is a protein-coding gene, is a top candidate gene for AD^29^. The SNPs in SORL1, DAPK1 and SORCS1 genes have shown significant association with hippocampal volume change, which is related to AD progression^42^. The VEGFA gene is associated with an increased risk of developing AD, as well as an accelerated cognitive decline^43^. The SNPs in APOE have also been related to neuroimaging measures in brain disorders such as MCI and AD^44^. The discovery of those SNPs by our method suggests that our method is able to identify the most relevant SNPs for AD diagnosis.

## Discussion

To sum up, we have presented a semi-supervised hierarchical feature and sample selection (ss-HMFSS) framework, in which both labeled and unlabeled data can be utilized to preserve the data manifold in the learning process. To validate the effectiveness of our method, we conducted experiments on AD diagnosis with both imaging and genetic data from ADNI cohort. Results showed that the proposed hierarchical scheme was able to gradually refine the feature and sample set in multiple steps, therefore leading to superior performances in AD vs. NC, MCI vs. NC, and pMCI vs. sMCI classifications.

In clinical applications, differentiating pMCI and sMCI is of great interest and importance. The results on pMCI vs. sMCI classification by our method in Table 3 indicate that the classification ability of our algorithm on this task is on par with that for MCI vs. NC classification (with an accuracy of 80.8% as compared to that of 80.1% for MCI vs. NC classification). Although the performance itself may not warrant highly accurate computerized diagnosis, we believe that the results by our method can aid physicians by providing a useful second opinion for reference.

Different from ROI-based MRI features, the dimensionality of the original SNP features is substantially higher. Since only a small set of genetic variants are directly related to AD^25^, using *a prior* knowledge from clinical studies to select only the most relevant SNPs may result in a more effective classification model learning.

We have use two different modalities, *i.e*., MRI and SNP, for three binary classification tasks. Although other modalities such as PET (positron emission tomography) and CSF (cerebrospinal fluid) are available for some subjects in the ADNI-1 dataset, the subjects in our experiment do not have complete data from each modality. Note that a subset of the population in our study may contain all data modality, yet the results from a smaller test set may be less informative and conclusive. We conjecture that, with the inclusion of more data modalities, the predictive performance of the trained diagnostic models can be further improved. Therefore, in future, we plan to utilize more data with additional modalities, *e.g*. PET and CSF, to help further improve diagnosis performance.

When examining the data used in this work, we noticed that the numbers of females and males are not homogeneous. Given that the available data are not quite abundant from a machine learning point of view, we decided to use all the data to train and validate our algorithm on sample and feature selection. This would help alleviate under-fitting in the iterative learning process if more data are needed than available. Regarding the ethnicity, over 90% of the subjects in this study are white, and the rest are mainly black or Asian. Therefore, this dataset may not be the first choice to study the correlation between AD and race. It has been reported that gender^45^,^46^ and race^47^,^48^ are important factors in AD studies. Studying the impact of gender or race on AD diagnosis would help further improve the algorithm development and diagnosis. For a more comprehensive study, dataset without demographic bias needs to be collected. In this work, we train a model without taking into account the gender or race information, and this is a current limitation. Nevertheless, the goal of this work is to introduce a generic machine learning framework, which can be readily applied for AD diagnosis. Future work is expected to address these aforementioned aspects. In addition, another limitation of our method is that it requires complete data from different modalities for each subject. Extending our method to handle incomplete data is our current ongoing work.

## Methods

### Data

The data in our experiments are from the ADNI-1 dataset (http://adni.loni.usc.edu). This dataset enrolls subjects who were 55–90 years old with study partners who can provide independent evaluations of functioning. The general inclusion/exclusion criteria for the enrolled subjects are the following:

- NC subjects: Mini-Mental State Examination (MMSE) scores between 24 and 30 (inclusive), a Clinical Dementia Rating (CDR) of 0, non-depressed, non-MCI, and non-demented.
- MCI subjects: MMSE scores between 24 and 30 (inclusive), a memory complaint, objective memory loss measured by education adjusted scores on Wechsler Memory Scale Logical Memory II, a CDR of 0.5, absence of significant levels of impairment in other cognitive domains, essentially preserved activities of daily living, and an absence of dementia.
- AD subjects: MMSE scores between 20 and 26 (inclusive), a CDR of 0.5 or 1.0, and meet the National Institute of Neurological and Communicative Disorders and Stroke and the Alzheimer’s Disease and Related Disorders Association (NINCDS/ADRDA) criteria for probable AD.

Specifically, in this study, we use 737 subjects whose MRI and SNP features are both available in the dataset. Among these subjects, 171 were diagnosed with AD, 362 were MCI patients, and the rest 204 subjects were NCs. Among the MCI patients, 157 of them were labeled as pMCI, and 205 were sMCI. The sMCI subjects were diagnosed previously as MCI patients but remained stable all the time, while pMCI refers to the MCI patients who converted to AD within a 24 months span. Table 5 summarizes the demographic information of the subjects in our experiments.

### Preprocessing

The data preprocessing follows the procedures as outlined in ref. 29. Specifically, for MRI data, the preprocessing steps included skull stripping^49^, dura removal, intensity inhomogeneity correction, cerebellum removal, tissue segmentation, and registration. The preprocessed images were then divided into 93 pre-defined ROIs based on the template in ref. 50, and the gray matter volume in these ROIs were calculated as MRI features. Note that the gray matter volumes were corrected for the total intracranial volume of each subject, in order to account for the body size variations in the population.

The SNP data were genotyped using the Human 610-Quad BeadChip^42^. According to the AlzGene database (www.alzgene.org), only SNPs that belong to the top AD gene candidates were selected after standard quality control (QC). The QC of SNP data included the following steps:

- Call rate check per SNP per subject.
- Gender check.
- Sibling pair identification.
- Hardy-Weinberg equilibrium test.
- Marker removal by the minor allele frequency.
- Population stratification.

After QC, the SNPs were imputed to estimate the missing genotypes, and the Illumina annotation information was used to select a subset of SNPs^51^. The dimensionality of the processed SNP data is 2098. Since this SNP feature dimension is much higher than that of MRI, we perform sparse feature learning^35^ on the training data to reduce the number of SNP features to the same dimension as the MRI features.

**Figure 4:**
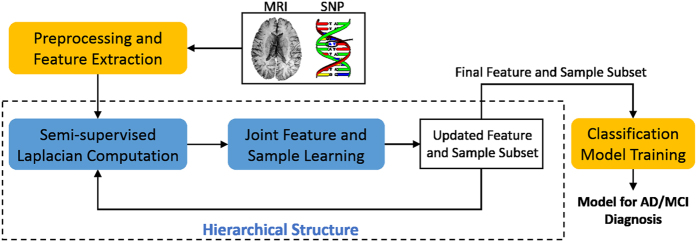
Framework of the proposed semi-supervised hierarchical multimodal feature and sample selection (ss-HMFSS) for AD and MCI diagnosis. The data are first preprocessed, and features are extracted from MRI and SNP, respectively. The MRI features and the preselected SNP features from both labeled and unlabeled data are used to exploit the data manifold via a Laplacian matrix computation. In a joint feature and sample selection learning framework, manifold preservation, feature selection, and sample selection are achieved. This learning process is performed in a hierarchical manner to gradually identify a set of the most discriminative features and samples, which are then used to train the classification model.

The framework of the proposed method is illustrated in Fig. 4. After features are extracted and preprocessed from the raw SNP and MRI data, we first calculate the graph Laplacian matrix to model the data structure, using the concatenated features from both labeled and unlabeled data. This Laplacian matrix is then used in the manifold regularization to jointly learn the feature coefficients and sample weights. In each hierarchy, the features are selected and weighted based on the learned coefficients, and the samples are pruned by discarding those with smaller sample weights. The updated features and samples are then forwarded to the next hierarchy for further selection, following the same process. In such a hierarchical manner, we gradually select the most useful features and samples to mitigate the adverse effect of data redundancy in the learning process. Finally, the selected features and samples are used to train classification models using SVM for AD/MCI diagnosis tasks. In the following, we explain in detail how the joint feature and sample selection works in each hierarchy.

Throughout this section, we use boldface uppercase letters to denote matrices (*e.g*., **X**), and boldface lowercase letters to denote vectors (*e.g*., **x**). All non-bold letters denote scalar variables. 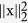 and 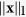 represent the squared Euclidean norm and the 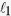 norm of **x**, respectively. The transpose of **X** is denoted as **X**^**Τ**^.

**Figure 5:**
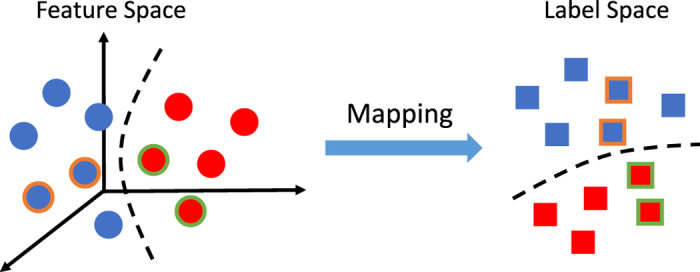
Illustration of the manifold regularization such that the neighborhood structures are preserved during the mapping from the feature space to the label space. Samples in different classes are denoted by different colors. The circles and squares with colored outline denote the neighbors.

Suppose we have *N*_*l*_ labeled training subjects with their class labels and the corresponding features from both MRI and SNP, denoted by 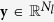, 
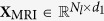
, and 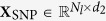, respectively. In addition, data from *N*_*ul*_ unlabeled subjects are also available, denoted as 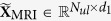, and 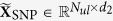. The goal is to utilize both labeled and unlabeled data in a semi-supervised framework, to jointly select the most discriminative samples and features for subsequent classification model training and prediction. Let 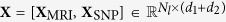 be the concatenated features of the labeled data, 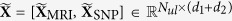 represent features of the unlabeled data, and 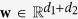 be the feature coefficient vector. Then, the objective function for this joint sample and feature learning model is given by


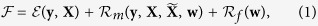


where 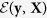 is the loss function defined on the labeled data, 
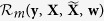
 is the manifold regularization term for labeled data as well as unlabeled data. The regularization term is based on the assumption that if two samples **x**_*p*_ and **x**_*q*_ are close to each other in their original feature space, after mapping into the new space (*i.e*., label space), their neighborhood structure should also be maintained, with an illustration given in Fig. 5. 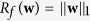 is the sparse regularizer for the purpose of feature selection, and only features with non-trivial coefficients in *w* are expected to be discriminative. In the following, we explain in detail how the loss function and the manifold regularization term are defined, and how the sample weights are incorporated.

### Loss Function

The loss function 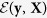 considers the weighted loss for each sample, and is given by


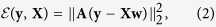


where 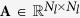 is a diagonal matrix, and each diagonal element denotes the weight for a data sample. Intuitively, a sample that can be more accurately mapped into the label space with minimal error is more desirable, comparatively, and thus it should contribute more to the classification model. The sample weights in **A** will be learned through optimization and the samples with larger weights will be selected to train the classifier.

### Manifold Regularization

The manifold regularization preserves the neighborhood structures for both labeled and unlabeled data when they are mapped from feature to label space:


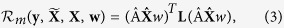


where 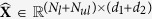 contains features of both labeled data **X** and unlabeled data 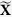. The Laplacian matrix 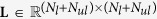 is given by **L** = **D** − **S,** where **D** is a diagonal matrix such that **D**(*p, p*) = ∑_*q*_**S**(*p, q*), and **S** is the affinity matrix with **S**(*p, q*) denoting the similarity between samples **x**_*p*_ and **x**_*q*_. **S**(*p, q*) is defined as


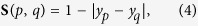


where *y*_*p*_ and *y*_*q*_ are the labels for **x**_*p*_ and **x**_*q*_. For the case of unlabeled data, *y*_*p*_ is a soft label for an unlabeled data sample **x**_*p*_, defined as


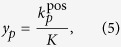


where 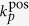 is the number of **x**_*p*_’s neighbors with positive class labels out of its *K* neighbors in total. Note that for an unlabeled sample, the nearest neighbors are searched only in the labeled training data, and the soft label represents its proximity to a target class. Using such definition, the similarity matrix **S** encodes relationships among both labeled and unlabeled samples.

The diagonal matrix 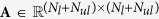 applies weights on both labeled and unlabeled samples. The elements in **A** are different for labeled and unlabeled data:


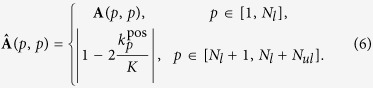


By this definition, if an unlabeled sample whose *K* nearest neighbors are relatively balanced from both positive and negative classes (*i.e*., 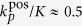), it is assigned a smaller weight, since this sample may not be discriminative enough in terms of class separation. The weights in **A** for the labeled data are to be learned in the optimization process.

### Objective Function

Taking into account the loss function, the manifold regularization, as well as the sparse regularization on features, the overall objective function is


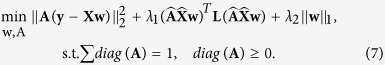


where the elements in **A** are enforced to be non-negative to assign physically interpretable weights to different samples. Also, the diagonal of **A** should sum to one, which makes the sample weights interpretable as probabilities, and ensures that sample weights will not be all zeros.

### Optimization

Since Eq. (7) is biconvex with respect to **w** and **A**, we employ an alternating optimization strategy to solve this problem, meaning that we split the objective function into two sub-problems and then solve them iteratively. When one unknown variable is fixed, the resulting sub-problem would be convex. In such a way, the original objective function can converge to the optimal point^52^. Specifically, we first fix **A** to find the solution of **w**, and then vice versa. When **A** is fixed, Eq. (7) becomes


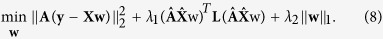


It is easy to verify that Eq. (8) is non-smooth, although it is convex, because of 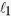-norm regularizer. One way to cope with this problem is to approach the original non-smooth objective function using a function which is smooth. Then this smooth objective function can be solved using standard fast algorithms. In this work, we resort to the widely used Accelerated Proximal Gradient (APG) method^53^ to solve Eq. (8).

In the second step, given a fixed **w**, the objective function in Eq. (7) reduces to


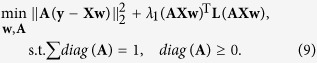


Note that since the unlabeled data are irrelevant to the original objective function in Eq. (7), we only need to optimize **A** via Eq. (9). Eq. (9) is convex with respect to **A**, and can be efficiently solved via quadratic programming^54^.

To this end, the discriminative features are identified by the significant values in **w**, and the poor samples are assigned lower weights in **A**. Therefore, those less useful features and samples can be discarded based on the values in **w** and **A**, which leads to a more compact yet effective subset of features and samples as compared with the original data. In addition, the learned coefficients in **w** can be used to weight the features, addressing their importance. This completes the first hierarchy. In the next hierarchy, the selected samples and updated feature sets are used similarly in the optimization of Eq. (7) to further refine the sample and feature sets. The entire process of the proposed method is summarized in Algorithm 1.


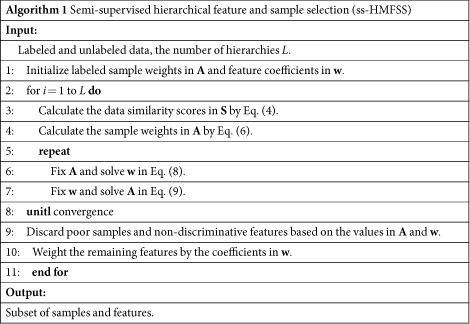


## Additional Information

**How to cite this article:** An, L. *et al*. A Hierarchical Feature and Sample Selection Framework and Its Application for Alzheimer’s Disease Diagnosis. *Sci. Rep.* **7**, 45269; doi: 10.1038/srep45269 (2017).

**Publisher's note:** Springer Nature remains neutral with regard to jurisdictional claims in published maps and institutional affiliations.

## Figures and Tables

**Table 1:** Comparison of classification performance with different feature modalities using the proposed method (in %).

Modality	AD vs. NC		MCI vs. NC		pMCI vs. sMCI	
	ACC	AUC	ACC	AUC	ACC	AUC
MRI	88.2^†^	93.6^†^	68.0^†^	73.4^†^	69.6^†^	74.2^†^
SNP	77.6^†^	85.5^†^	73.3^†^	83.1^†^	79.4*	86.3^†^
MRI + SNP	**92.4**	**97.4**	**80.1**	**87.2**	**80.8**	**88.3**

Symbol ^†^ indicates *p* < 0.01 in the *t*-test as compared to the proposed method, and * means *p* < 0.05.

**Table 2:** Comparison of classification performance of feature and sample selection (in %).

Selection	AD vs. NC		MCI vs. NC		pMCI vs. sMCI	
	ACC	AUC	ACC	AUC	ACC	AUC
Sample only	88.9^†^	95.1^†^	74.9^†^	80.7^†^	76.2^†^	84.5^†^
Feature only	91.3*	96.4*	77.3^†^	85.0^†^	78.1^†^	85.9^†^
Sample + Feature	**92.4**	**97.4**	**80.1**	**87.2**	**80.8**	**88.3**

Symbol ^†^ indicates p < 0.01 in the *t*-test as compared to the proposed method, and * means *p* < 0.05.

**Table 3:** Comparison of classification performance by six different methods (in %).

Method	AD vs. NC				MCI vs. NC				pMCI vs. sMCI			
	ACC	SPE	SEN	AUC	ACC	SPE	SEN	AUC	ACC	SPE	SEN	AUC
no FS (MRI only)	88.3^†^	81.9^†^	92.2^†^	94.1^†^	72.5^†^	80.7^†^	42.9^†^	72.0^†^	68.4^†^	59.4^†^	75.9^†^	73.2^†^
no FS (SNP only)	77.3^†^	75.3^†^	80.6^†^	85.3^†^	74.8^†^	83.2^†^	36.1^†^	74.1^†^	73.1^†^	67.7^†^	80.8^†^	79.2^†^
no FS (MRI + SNP)	87.5^†^	81.6^†^	90.3^†^	95.6^†^	73.8^†^	85.1^†^	53.6^†^	80.6^†^	74.7^†^	64.5^†^	78.8^†^	83.4^†^
Laplacian score^33^	87.7^†^	83.3^†^	90.9^†^	94.3^†^	73.9^†^	85.1^†^	53.0^†^	81.4^†^	76.6^†^	65.2^†^	78.8^†^	77.6^†^
Pearson correlation^34^	87.8^†^	84.9^†^	89.7^†^	94.6^†^	73.7^†^	83.6^†^	53.3^†^	79.1^†^	77.1^†^	66.9^†^	82.5^†^	78.6^†^
*t*-test^31^	87.8^†^	84.5^†^	90.3^†^	94.2^†^	73.1^†^	84.4^†^	53.1^†^	80.4^†^	76.0^†^	65.5^†^	78.3^†^	78.1^†^
Fisher score^32^	88.8^†^	85.9*	91.4^†^	94.9^†^	73.6^†^	84.9^†^	57.4^†^	82.1^†^	76.7^†^	68.0^†^	83.8^†^	78.4^†^
LASSO^35^	89.2^†^	83.6^†^	90.7^†^	95.8^†^	74.7^†^	**87.3**	57.6	83.2^†^	76.3^†^	68.1^†^	83.0^†^	77.9^†^
RIML^17^	89.4^†^	85.0*	90.2^†^	94.9^†^	75.6^†^	85.2^†^	56.4^†^	83.9^†^	77.0^†^	68.6^†^	84.4*	84.9^†^
HMFSS	90.8^†^	84.1^†^	94.3^†^	97.1*	77.9*	84.0^†^	65.9^†^	85.4^†^	78.6^†^	69.2*	84.9*	85.7^†^
ss-HMFSS	**92.4**	**86.0**	**95.9**	**97.4**	**80.1**	85.5*	**67.7**	**87.2**	**80.8**	**71.5**	**85.4**	**88.3**

Symbol ^†^ indicates *p* < 0.01 in the *t*-test as compared to the best method, and * means *p* < 0.05.

**Table 4:** Most selected SNP features for AD diagnosis.

Gene name	SNP name
CTNNA3	rs10740220, rs10997232
SORL1	rs2298525, rs4420280
SORCS1	rs11814145
DAPK1	rs913782
VEGFA	rs833069
APOE	rs429358

**Table 5:** Demographic information of the 737 subjects used in this work from the ADNI-1 dataset.

Diagnosis	# of Subject	Age	Gender (M/F)	Education	MMSE
AD	171	75.5 ± 7.7	94/77	14.5 ± 3.7	23.7 ± 1.9
pMCI	157	74.8 ± 7.0	95/62	16.1 ± 2.5	26.9 ± 1.8
sMCI	205	75.1 ± 7.6	137/68	15.8 ± 3.1	27.4 ± 1.6
NC	204	76.1 ± 4.9	112/92	15.9 ± 3.0	29.1 ± 1.0
